# A Case of Bowen’s Disease and Small-Cell Lung Carcinoma: Long-Term Consequences of Chronic Arsenic Exposure in Chinese Traditional Medicine

**DOI:** 10.1289/ehp.7200

**Published:** 2004-12-20

**Authors:** Linda Lee, Gwyn Bebb

**Affiliations:** ^1^University of Alberta, Faculty of Medicine, Edmonton, Alberta, Canada; ^2^Tom Baker Cancer Centre, Calgary, Alberta, Canada

**Keywords:** arsenic, Bowen’s disease, case report, Chinese traditional medicine, chronic toxicity, small-cell lung carcinoma

## Abstract

Chronic arsenic toxicity occurs primarily through inadvertent ingestion of contaminated water and food or occupational exposure, but it can also occur through medicinal ingestion. This case features a 53-year-old lifetime nonsmoker with chronic asthma treated for 10 years in childhood with Chinese traditional medicine containing arsenic. The patient was diagnosed with Bowen’s disease and developed extensive-stage small-cell carcinoma of the lung 10 years and 47 years, respectively, after the onset of arsenic exposure. Although it has a long history as a medicinal agent, arsenic is a carcinogen associated with many malignancies including those of skin and lung. It is more commonly associated with non–small-cell lung cancer, but the temporal association with Bowen’s disease in the absence of other chemical or occupational exposure strongly points to a causal role for arsenic in this case of small-cell lung cancer. Individuals with documented arsenic-induced Bowen’s disease should be considered for more aggressive screening for long-term complications, especially the development of subsequent malignancies.

A 53 year-old nonsmoking Chinese man with a long-standing diagnosis of asthma presented with a 1-week history of worsening shortness of breath, productive cough, and pleuritic chest pain. Chest X ray confirmed right-middle lobe consolidation suggestive of pneumonia. His symptoms resolved with a 5-day course of antibiotics, but a follow-up chest X ray demonstrated worsening of the consolidation. Bronchoscopy revealed a right middle-lobe tumor, and the biopsy was positive for small-cell lung carcinoma.

The patient was born in Hong Kong and immigrated to Canada around 1990 at 40 years of age. According to his medical history, at 6 years of age he was diagnosed with asthma, which was treated with Chinese traditional medicine (CTM) pills known to contain arsenic until he was 16 years of age, when he was diagnosed with Bowen’s disease. In 1993, a skin biopsy of a lesion on his right elbow was positive for squamous cell carcinoma *in situ*. He had never smoked cigarettes, and he drank alcohol only once a month. He had no other known exposures to arsenic through drinking water, diet, or occupation.

When seen by the medical oncologist, he complained of a cough that produced clear sputum and dull pain over the right costal margin, but he denied constitutional symptoms. A physical exam revealed an afebrile Asian male with no palpable lymphadenopathy. Chest examination was consistent with right middle lobe consolidation. The liver edge was palpable 3 cm below the costal margin but was neither nodular nor tender. Multiple 3–8 cm scaly, purple-brown, well-demarcated tear-drop–shaped patches were distributed over the skin of his trunk and extremities; these patches were consistent with his history of Bowen’s disease. His neurologic examination was normal.

Computed tomography of the patient’s chest and abdomen confirmed a right hilar mass completely obstructing the right middle-lobe bronchus with distal pneumonitis and large mediastinal nodes. Multiple hypodense liver lesions suggestive of metastatic disease were also found.

A diagnosis of extensive stage small-cell lung cancer was made. The patient entered a phase III clinical trial comparing cisplatin and etoposide to cisplatin and irinotecan and was randomized to the latter arm. Because more than 35 years had passed since the cessation of his known arsenic exposure, direct testing to confirm a diagnosis of arsenic exposure would probably have served little purpose. He achieved a complete remission but died of complications of progressive disease 10 months later.

## Discussion

Arsenic, a naturally occurring metal, is best known as a poison and can generate both acute and chronic toxicity. Exposure can occur through air, water, soil, and food. Less well known is that arsenic has been used medicinally to treat a variety of illnesses since the times of Hippocrates and Galen. In the 18th and 19th centuries, Fowler’s solution (containing 1% arsenic trioxide) was prescribed to treat asthma, psoriasis, syphilis, and chronic myelogenous leukemia (Waxman and Anderson 2001). Although the association of inorganic arsenic intake and malignancy was first documented in 1887 (Hutchison 1887), Fowler’s solution continued to be popularized as a health tonic and was listed in the British *Pharmaceutical and Therapeutic Products* handbook as recently as 1958 (Ratnaike 2003).

With the advent of other pharmaceuticals and the discovery of the complications of chronic arsenic exposure, therapeutic arsenic use has declined. By the mid-1990s, the only indication for arsenic was in the treatment of trypanosomiasis (Waxman and Anderson 2001). However, intravenous arsenic trioxide has recently been shown to induce remission in patients with acute promyelocytic leukemia (Soignet et al. 1998), prompting a resurgence in its therapeutic use. In 2000, the U.S. Food and Drug Administration (FDA) approved the drug Trisenox (arsenic trioxide; Cell Therapeutics, Inc., Seattle, WA, USA) only 3 years after studies were initiated (FDA 2001).

In our patient, chronic arsenic exposure occurred by consuming Chinese herbal anti-asthmatic preparations in Hong Kong, most likely between 1956 and 1966. Although these medicines were widely available throughout Asia, their use was first described in Singapore by Tay and Seah (1975). They identified preparations containing high concentrations of inorganic arsenic ranging from 25 to 107,000 mg/L, with six of these preparations having been imported from Hong Kong.

Arsenic continues to be either a main constituent or a contaminant in many traditional and herbal medicines. An analysis of Asian traditional medicines by Garvey et al. (2001) revealed that 4 of the 54 (7.4%) sampled pills would result in a daily arsenic dosage of > 0.1 mg/day, whereas other pills contained significant quantities of mercury or lead. Containing daily dosages of arsenic that ranged from 0.140 to 16.1 mg, these pills were indicated for the treatment of asthma, headache, fever, and children’s ailments and to clear the kidneys and lungs (Garvey et al. 2001). Although manufactured in Southeast Asia, two of the pills were purchased in the United States (Garvey et al. 2001). In recent decades, there has been growing interest and availability of traditional Asian medicines. Currently, the FDA has not imposed any standard limits for arsenic in food or medicine except in animals treated with veterinary drugs [Agency for Toxic Substances and Disease Registry (ATSDR) 2004].

The toxicity of arsenic depends on its chemical state. Inorganic arsenic in its trivalent form is more toxic than pentavalent arsenic (Hughes 2002). By binding to thiol or sulfhydryl groups on proteins, As(III) can inactivate over 200 enzymes (Abernathy et al. 1999; Hughes 2002). This is the likely mechanism responsible for arsenic’s widespread effects on the liver, lungs, kidneys, spleen, gastrointestinal tract, and keratin-rich tissues. As(V) can replace phosphate, which is involved in many biochemical pathways resulting in the depletion of compounds such as adenosine-5′-triphosphate (ATP) (Hughes 2002). Increased levels of reactive oxidants in plasma (Wu et al. 2001) and markers of oxidative damage in arsenic-related skin conditions (Matsui et al. 1999) suggest that long-term damage from chronic arsenic exposure is mediated through the generation of reactive oxygen species.

Investigations of the effects of arsenic in animals have been problematic (Wang JP et al. 2002), and it is uncertain whether inorganic arsenic itself or resultant methylated metabolites that form *in vivo* are responsible for the carcinogenic effect. The few studies that have been successful, however, confirm arsenic’s carcinogenicity. Arsenic is likely a cocarcinogen that inhibits DNA repair and enhances the activity of other directly genotoxic agents (Andrew et al 2003; Beyermann 2002; Rossman et al. 2002). The cellular response to arsenic exposure seems to be concentration dependent. At high concentrations (> 50 μM), arsenic is able to induce an apoptotic response *in vitro*, a phenomenon probably exploited in its use to treat leukemia (Jimi et al 2004; Lunghi et al 2004). At lower concentrations (< 25 μM), evidence of genomic stress can be observed in the form of nuclear accumulation of p53, but apoptosis is not generally seen (Dong 2002). Environmental exposure to arsenic in a chronic low-dose manner likely leads to the gradual accumulation of genomic damage without apoptosis. Other observations suggest that the role of arsenic as a cocarcinogen may be mediated by inhibition of DNA repair and increased expression of cyclin D1 (Vogt and Rossman 2001). Differences in the expression pattern of p53 have also been attributed to the down-regulation of gene expression by alteration of promoter methylation status (Mass and Wang 1997). Arsenic has been shown to modulate cell signaling by inducing mitogen-activated protein kinases to change gene expression (Beyersmann 2002; Yang and Frenkel 2002).

Diagnosis of arsenic intoxication is often difficult because clinical presentation varies depending on route of exposure, chemical form, dose, and time elapsed since exposure. Furthermore, because arsenic affects multiple systems, poisoning can present with a wide variety of signs and symptoms. In acute arsenic poisoning, initial symptoms are gastrointestinal in nature due to the direct toxic effect of arsenic on intestinal epithelial cells. Clinical features include colicky abdominal pain, nausea, vomiting, bloody or rice-water diarrhea, and excessive salivation. Other manifestations include acute psychosis, cardiomyopathy, pulmonary edema, renal failure, skin rash, anemia, and encephalopathy (Ratnaike 2003). Quantitative studies can be performed on blood and urine in acute arsenic poisoning to confirm a suspected diagnosis. Because arsenic is cleared from blood within 10 hr (Hindmarch 2002), a urine arsenic level is usually more useful in cases of recent ingestion within 1–3 days (Buchet et al. 1981). Residual traces of arsenic in hair and nail samples may confirm arsenic exposure but can be subject to external contamination and cannot reliably date time of exposure (Hindmarch 2002). Presence of anemia, leukopenia, thrombocytopenia, or eosinophilia on complete blood count, basophilic stippling on the peripheral smear, or elevated liver transaminases is consistent with arsenic exposure but is not specific.

Like acute arsenic poisoning, the clinical features of chronic arsenic exposure are multi-systemic. Symptoms include malaise, weakness, decreased appetite, weight loss, and a sensory peripheral neuropathy that progresses to glove and stocking anesthesia (Ratnaike 2003). However, the hallmark of long-term arsenic exposure involves cutaneous changes such as hyperkeratosis, hyperpigmentation, Mee’s lines on nails, and malignant skin changes including Bowen’s disease, squamous-call carcinoma, and basal-cell carcinoma (Centano et al. 2002; Wong SS 1998). Although cutaneous changes develop slowly over time (up to 3–7 years for pigmentation changes and keratoses and up to 40 years for skin cancer), they may occur after lower doses than those causing neuropathy or anemia (ATSDR 2004). Studies on populations with chronic exposure to arsenic through drinking water show an association with increased cardiovascular disease (Tseng et al. 2003; Wang CH et al. 2002), peripheral vascular disease (Wang CH et al. 2002; Wang et al. 2003; Yu et al. 2002), cerebrovascular disease (Wang CH et al. 2002), respiratory disease (Milton and Rahman 2002), and diabetes (Wang et al. 2003). The most serious long-term consequence of arsenic exposure is increased risk for malignancy. Arsenic is now a recognized carcinogen associated with increased incidence of skin, lung, liver, bladder, and kidney malignancies (Chen et al. 1992).

A positive dose–response relationship between arsenic exposure and its chronic health effects has been observed. In a large population study in West Bengal, the prevalence of both skin lesions and hyperpigmention increased with the concentration of arsenic in drinking water (Mazumder et al. 1998). For levels of arsenic > 0.80 mg/L, the prevalence of skin lesions and hyperpigmentation in males was 10.7 and 22.7 per 100, respectively (Mazumder et al. 1998). A similar trend was noted in a Taiwanese study; for arsenic levels > 0.60 mg/L, the prevalence of skin cancer by 60 years of age was 92.0 in 1,000 (Tseng et al. 1968). In a recent review of the dose–response relationship between arsenic consumption through drinking water and its adverse health effects, Yoshida et al. (2004) noted that skin lesions are the most sensitive feature and often the earliest nonmalignant effect of arsenic exposure. Although a dose–response relationship between arsenic and certain malignancies including lung cancer was first identified in 1989 on the basis of a maximum 22-year latency period (Wu et al. 1989), more recent data have quantified that only arsenic exposure levels > 0.64 mg/L are associated with a significant increase in lung cancer mortality (Guo 2004).

Medicinal arsenic ingestion typically results in prolonged toxic exposure at doses higher than those present in contaminated water (Garvey et al. 2001; Tay and Seah 1975). Because both types of exposure involve trivalent arsenic and occur through the same mechanism of oral consumption followed by gastrointestinal absorption, it is possible that epidemiologic data from studies of drinking-water exposure may be applied to medicinal arsenic exposure. In 1998, case reports of three patients with chronic arsenic poisoning from CTMs in Singapore document that all three had cutaneous changes including basal-cell carcinoma and squamous-cell carcinoma, one had lung cancer, and one had liver cancer (Wong ST et al. 1998).

Another case review of 17 patients from Singapore selected for arsenic-induced cutaneous changes found that 15 were exposed through CTMs (Wong SS et al. 1998). All the patients had Bowen’s disease that developed after a long average latency period of 39 years. Since 1995, CTMs containing > 5 mg/L inorganic arsenic have been banned in Singapore (Wong SS et al. 1998).

Skin lesions generally precede the onset of internal malignancies. In a study of patients who took Fowler’s solution (containing 1% arsenic trioxide) during 1945–1969, approximately 50% had arsenic-related skin changes (Cuziek et al. 1982). A follow-up report 10 years later demonstrated excess bladder cancer mortality in the subgroup of patients with skin changes (Cuziek et al. 1992). In a study in Japan, Miki et al. (1982) reported that of 31 patients with Bowen’s disease and increased arsenic levels through drinking water, 10 had invasive skin cancers and 10 had internal malignancies, including 7 patients with pulmonary cancers. The authors hypothesized a timeline in which exposure to arsenic was followed by Bowen’s disease within 10 years, invasive skin cancers after 20 years, and pulmonary cancers after 30 years. Given the long period for the development of arsenic-induced malignancy, it may be too early to see cases arising from areas currently affected by contaminated drinking water such as West Bengal, Bangladesh, and China. In our patient, exposure likely started at approximately 6 of age, with his Bowen’s disease and pulmonary cancer diagnosed 10 and 47 years later, respectively.

Multiple studies have demonstrated that arsenic exposure is a documented risk factor for the development of lung carcinoma. This was best shown in a study in Nakajo, Japan (Nakadaira et al. 2002), in which some residents were exposed to well water with inorganic arsenic levels as high as 400 mg/L during 1954–1959. Of 454 inhabitants who underwent medical examinations in 1959,93 (20.5%) were diagnosed as having chronic arsenic poisoning on the basis of physical signs including cutaneous changes. Twenty-nine years after the exposure was terminated, exposed male patients demonstrated an excess mortality rate from lung cancer: the ratio of observed deaths to expected deaths from lung cancer was 7.0:0.64. Although arsenic exposure is more commonly associated with non–small-cell lung cancer, small-cell carcinoma incidence was also increased when compared with control groups, thereby supporting a causal relationship between arsenic and small-cell lung cancer. However, smoking was a confounding factor that was not addressed in the study design.

Bowen’s disease (squamous-cell carcinoma *in situ*), which can arise as a consequence of both arsenic and exposure to ultraviolet (UV) radation, seems a natural platform from which to study carcinogenic changes. A large population-based Danish cohort study confirmed that patients with Bowen’s disease have an excess risk of nonmelanomatous skin cancer and found a 2-fold increase in the risk of lung cancer in male patients with Bowen’s disease on sun-protected areas (Jaeger et al. 1999). The different mutagenic mechanisms associated with arsenic compared with those of other genotoxic agents have been reflected in mutational spectra of specific genes. Subtle differences have been noted in the mutation spectrum of UV-induced Bowen’s disease, in which point mutations are common, in contrast to arsenic-induced skin lesion, for which few *p53* mutations were observed (Castren et al. 1998; Hsieh et al. 1994). Whether such observations can be translated from cutaneous lesions to bronchial neoplasms is unclear, but it seems likely that a different mutational spectrum may be seen in arsenic-induced versus smoking-induced small-cell lung cancers.

Small-cell lung cancer is an aggressive tumor that metastasizes early. Patients often present with extensive disease, which has a poor prognosis. It is extremely rare in young nonsmokers, so such a diagnosis should provoke a search for other risk factors. In the present case, the only risk factor was a remote 10-year history of arsenic ingestion through CTMs. Physical examination revealed the presence of cutaneous lesions that had been previously biopsied to show the presence of Bowen’s disease. Although peripheral neuropathy was absent, we felt that his cutaneous and lung neoplasms served to indirectly confirm his history of remote chronic arsenic exposure.

## Conclusion

Chronic arsenic toxicity is a clinical diagnosis. It can be difficult to elicit a clear history of exposure either to contaminated food or well water, or through occupational exposure. However, it is a diagnosis that should be considered if there is a clear history of traditional or herbal medication use, particularly for the treatment of asthma, psoriasis, or syphilis. Moreover, chronic arsenic toxicity should be suspected in anyone presenting with cutaneous changes such as hyperkeratosis, hyperpigmentation, Mee’s lines on nails, or malignant skin changes such as Bowen’s disease with or without concomitant peripheral neuropathy.

Arsenic is a recognized etiologic factor in Bowen’s disease and a known risk factor for lung cancer. In our patient, it is likely that each condition developed independently following arsenic exposure, with skin pathology preceding lung cancer. Although a unifying pathophysiologic mechanism remains to be elucidated, patients with a history of arsenic exposure or ingestion of antiasthmatic CTMs require additional vigilance for signs of skin changes that may herald other malignancies. As chronic arsenic exposure through contaminated drinking water continues in many areas of the world, a large population may be at risk for latent malignancy, particularly if skin changes have already been noted. Because the role of chemopreventative approaches in these patients remains to be proven, such individuals should be considered candidates for chemoprevention trials.
